# “There Is Nothing I Cannot Achieve”: Empowering Latin American Women Through Agricultural Education

**DOI:** 10.3389/fpsyg.2022.902196

**Published:** 2022-06-24

**Authors:** Judith L. Gibbons, Zelenia Eguigure-Fonseca, Ana Maier-Acosta, Gladys Elizabeth Menjivar-Flores, Ivanna Vejarano-Moreno, Alexandra Alemán-Sierra

**Affiliations:** ^1^Department of Psychology, Saint Louis University, St. Louis, MO, United States; ^2^Decanatura Académica, Zamorano University, Tegucigalpa, Honduras; ^3^Recursos Humanos, Zamorano University, Tegucigalpa, Honduras

**Keywords:** agricultural education, women, machismo, empowerment, self-efficacy

## Abstract

Higher education, a key driver of women’s empowerment, is still segregated by gender across the world. Agricultural higher education is a field that is male-dominated, even though internationally women play a large role in agricultural production. The purpose of this study was to understand the experience, including challenges and coping strategies, of women from 10 Latin American countries attending an agricultural university in Latin America. The participants were 28 women students with a mean age of 20.9 ± 1.8 years. Following informed consent and assurance of confidentiality, four focus group sessions (one for each year of study with a mean duration of 81 min) were conducted in Spanish. The central question was, “what has been your experience at the university?” Sessions were recorded and transcribed. Thematic coding was performed independently by two teams of researchers (from Latin America and North America), with the resulting schemas combined through mutual discussion. Member checking, auditing, and reflexivity contributed to trustworthiness of the process. Students reported that the personal qualities needed for success included determination, persistence, and self-efficacy. Many described an empowerment process, including increased discipline and self-efficacy from the first to fourth year of study. University life encompassed six themes: university structure and discipline (part of the exosystem), two supportive microsystems (friends and classmates and institutional support) as well as three challenges (academics, peers, and machismo). Cultural influences instantiated in students’ daily experiences included familism, machismo, and religious faith. Students anticipated futures involving further education and contributions to society. We conclude that higher education in agriculture can serve as an effective means of empowering women to feed the world.

## Introduction

Education is the key driver of women’s empowerment. Education reduces women’s poverty (McCarthy, 2015), allows them better employment opportunities (Spierings et al., 2010), empowers them to have the number of children they desire (Poelker and Gibbons, 2018), improves women’s health (Alsan and Cutler, 2013), and increases their political participation (Fanny and Oluwasanumi, 2014; Bird, 2019). Despite decades of efforts to achieve equality, higher education is still segregated by gender across the world (Charles and Bradley, 2009). One educational field dominated by men is agricultural and rural development (Acker, 1999; Dunne et al., 2021). In a 1990 study of agricultural education in Mexico, Central America, and the Caribbean, only 10% of students, professors, and graduates were women (Macías-López, 1990). Although women’s access to agricultural education has undoubtedly increased since 1990, women continue to be under-represented (Acker, 1999; Dunne et al., 2021). The purpose of the present study was to document the experiences of women attending an agricultural university where they represent only one third of the student body and to understand both the factors that impede their growth and the factors that empower them.

Women play major roles in farming and agriculture around the world. Although their participation varies by region, crop, ethnicity, and economic status, women represent approximately 43 percent of the agricultural labor force internationally (FAO, 2011). Compared to men they have less access to resources such as land ownership, technology, and control over production and sales (FAO, 2011). The Women’s Empowerment in Agriculture Index (WEAI) assesses women’s agricultural empowerment in five domains, including, “(1) decisions about agricultural production, (2) access to and decision-making power about productive resources, (3) control of use of income, (4) leadership in the community, and (5) time allocation” (Alkire et al., 2013, p. 71).

The education of women in agriculture and rural development has been implemented most broadly through extension courses rather than through formal university degree programs. Extension is an educational process for rural populations that aims to improve their livelihoods rather than provide a degree or certification (Oakley and Garforth, 1985). Extension varies widely and is adapted to local crops, climate, and culture. As is the case with formal agricultural education, compared to their male counterparts, women have significantly less access to extension (Peterman et al., 2014) and programs may be designed for and directed toward men, ignoring the constraints on women’s participation (Stern et al., 2016). Those gender inequities are important and far-reaching because extension education is an effective means of empowering women in agriculture. A study in Uganda concluded that “Overall, our findings suggest that extension programs aimed at providing women with information—thereby addressing intrahousehold information asymmetries—may be a first-best means of empowering women in agriculture” (Lecoutere et al., 2019, para. 1). An intervention in Bangladesh that included agricultural extension education improved women’s WEAI scores (Quisumbing et al., 2022). Women’s empowerment in agriculture, primarily through extension education and development projects, can also lead to increased food security (Murugani and Thamaga-Chitja, 2019; Ogunnaike et al., 2019), better nutrition for mothers and their children (Malapit et al., 2015; Malapit and Quisumbing, 2016), dietary diversity (Malapit and Quisumbing, 2016), and improved growth in children (i.e., increased height for age scores) (Malapit et al., 2015). In addition, women’s empowerment in agriculture mitigates some of the negative consequences of low diversity in crops (Malapit et al., 2015) and can impel a shift from subsistence farming to commercialization (Uwineza et al., 2021). Further education, especially higher education, in agriculture could potentially empower women in all five domains measured by the WEAI – to make decisions about production, gain access to knowledge and other resources, control income, serve as community leaders, and allocate their time more effectively.

The objective of the United Nations’ sustainable development goal 5 (SDG5) is achievement of gender equality. The provision of agricultural education to women can contribute to that goal, as well as to the elimination of poverty (SDG1), zero hunger (SDG2) and good health and well-being (SDG3). Those goals are mutually reinforcing, with education being a fundamental means to achieve them.

Although Latin America is extremely diverse with respect to geography, language, culture, economic condition, and gender roles, virtually all Latin American countries have a history of colonization, high indices of Christianity, and cultures that highlight the family as the basic unit of society. In cross-cultural studies most countries of Latin America were deemed to be collectivistic (Hofstede, 2001; Krys et al., 2022). As collectivism was unpacked and Latin American cultures studied more extensively, Latin Americans have been found to endorse collectivistic values toward in-groups, and to express their emotions openly, especially socially-engaged emotions that promote interdependence (GLOBE, 2020; Krys et al., 2022; Salvador et al., 2022). Latin American countries also evidence a wide gender-gap, especially in the domain of political empowerment and to a lesser extent economic participation and opportunity (World Economic Forum, 2021). The World Economic Forum (2021) estimates that at current rates of change, it will take 68.9 years to reach gender parity. The vulnerability of women to gender-related violence, climate change, and poverty also threatens their well-being (Bott et al., 2012; Goh, 2012; Bradshaw et al., 2019).

Latin America is a critical region of the world for women in agriculture because they are severely underrepresented; women make up about 20% of the agricultural labor force in Latin America (FAO, 2011). Moreover, they are disadvantaged, compared to their male counterparts, with respect to resources such as land ownership, access to new technologies, as well as formal and extension education (Deere and Leon, 2003; FAO, 2011).

The present study was designed to document and understand the experience, including challenges and ways of meeting those challenges, of women from 10 Latin American countries attending an agricultural university in Latin America. A phenomenological approach using focus groups allowed a deep understanding of the essence of women’s experience in agricultural higher education. What obstacles did the women confront and what were their sources of strength and adaptation?

Bronfenbrenner’s ecological theory was applied as a conceptual framework to structure the findings. According to that theory the environment of the individual person consists of microsystems (those settings in which the individual directly participates) as well as exosystems, mesosystems, and macrosystems (Bronfenbrenner, 1977). Bronfenbrenner added a time dimension, the chronosystem, in a later formulation (Bronfenbrenner, 1986). In addition, like Vélez-Agosto et al. we see culture, not as separate and distal from the settings of everyday life, but rather embedded in everyday practices (Vélez-Agosto et al., 2017). The content of the women’s experiences, the specific challenges and coping strategies represented in codes and themes, were revealed using a bottom-up thematic coding process.

## Materials and Methods

### Participants

The participants were 28 women students attending an agricultural/rural development university in Central America. Their mean age was 20.9 ± 1.8 years of age. They represented all four years of study and the three scholarship statuses (full, partial and none). See Table 1.

**TABLE 1:** Participant Information.

Pseudonym	Year in university	Country of origin	Age	Scholarship status	Field of study
Ana	First	Honduras	23	Partial	Agricultural Engineering
Aquira	First	Ecuador	19	None	Environment and Development
Cinthia	First	El Salvador	22	Full	Agricultural Engineering
Diana	First	Dominican Republic	19	Full	Food Science and Technology
Estephany	First	Honduras	19	Partial	Agribusiness Management
Gaby	First	Panama	20	Full	Food Science and Technology
Ruth	First	Honduras	19	Partial	Agricultural Engineering
Rubi	First	Haiti	22	Full	Environment and Development
Sandy	First	El Salvador	24	Full	Food Science and Technology
Sarai	First	Nicaragua	19	None	Agricultural Engineering
Aisha	Second	Ecuador	20	Full	Agricultural Engineering
Amatista	Second	El Salvador	21	Full	Food Science and Technology
Elizabeth	Second	Ecuador	20	Partial	Food Science and Technology
Jessy	Second	Haiti	21	Total	Agribusiness Management
Scarleth	Second	Guatemala	20	None	Food Science and Technology
Soraya	Second	Ecuador	19	Partial	Agribusiness Management
Gloria	Third	Honduras	22	Total	Food Science and Technology
Hazul	Third	Panama	20	Total	Agricultural Engineering
Jessica	Third	Dominican Republic	25	Total	Environment and Development
Juliana	Third	Dominican Republic	20	Total	Environment and Development
Julieta	Third	Honduras	22	None	Food Science and Technology
Rosalina	Third	Guatemala	20	None	Agribusiness Management
Ana	Fourth	Honduras	19	Partial	Agricultural Engineering
Jeni	Fourth	Guatemala	21	None	Agricultural Engineering
Juana	Fourth	Dominican Republic	22	Total	Agricultural Engineering
Silvana	Fourth	Colombia	24	None	Environment and Development
Tutis	Fourth	Guatemala	20	Partial	Agricultural Engineering
Victoria	Fourth	Ecuador	22	Partial	Food Science and Technology

*Because two students chose the pseudonym Ana, they are identified in the text by the year in school.*

### Context

The university where the study took place enrolls students from many parts of Latin America and the Caribbean. Although it was founded in 1942, the first women (6) were admitted in 1981 and represented 3.9% of the class. In 2018, the year the data were collected, there were 397 women students at the university, representing 33.4% of the student body.

### Procedure

After approval by the IRB of Hope College and the research office of Zamorano University, a random sample of 45 female students, drawn from the enrollment list and stratified by year in school and scholarship status, was invited to attend focus group sessions. Except for 17 students with scheduling conflicts, all those invited agreed to participate. Both informed consent and a promise of confidentiality (to protect other participants) were obtained. The research team had developed the focus group protocol and questions in meetings in which we also reviewed the procedures for conducting focus groups and coding qualitative data. Moderators of the focus groups were professional women administrators and staff from the agricultural university. Four focus group sessions, one for each year of study, were conducted in Spanish in a conference room at the university. The central question was, “what has been your experience at the university?” and follow-up questions queried about participants’ goals, challenges, perceived sources of support, and advice to women students arriving at the university. The mean duration of the sessions was 81 min. All sessions were recorded and transcribed in the original Spanish.

### Coding

**FIGURE 1:**
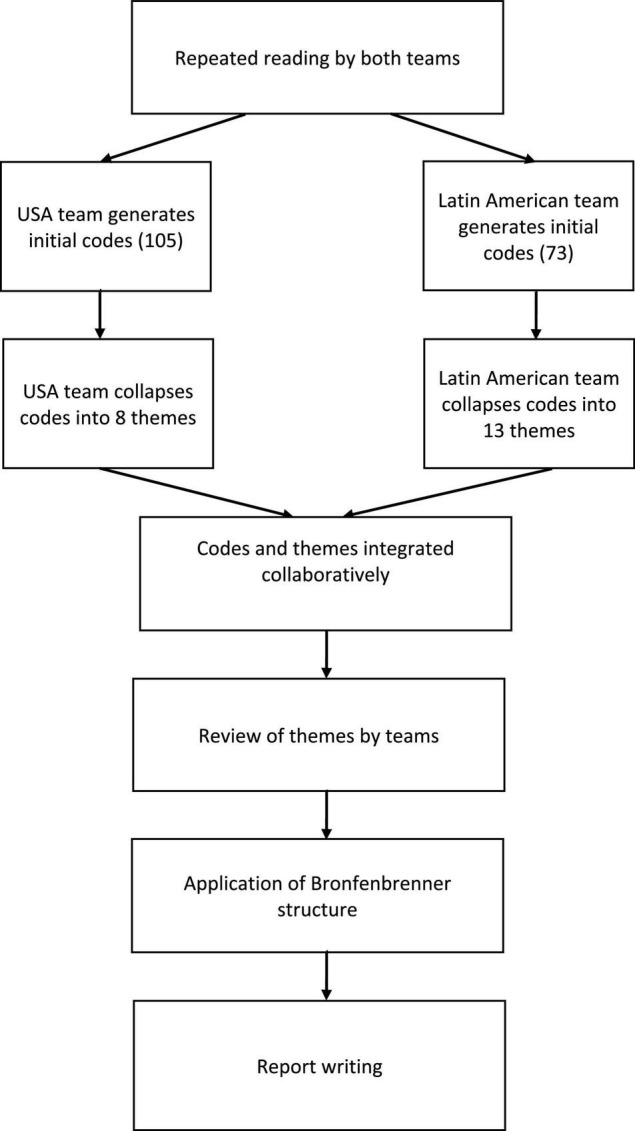
The coding process.

Thematic coding (in Spanish) was performed independently by two teams of researchers, one team of six women from Latin America and a second team of three women from the United States, all fluent in Spanish. We applied thematic coding according to Braun and Clarke’s (2006) method and elements of consensual qualitative research (Hill, 2012) in that the coding was done in teams. Each team worked independently to identify significant statements and repeating ideas labeled as codes. Those codes were recorded throughout the transcripts and then grouped into themes. Within teams, discrepancies were reconciled by discussion. Each team developed a hierarchical schema of how women students achieved success at the university. Those schemas from the two teams were then reviewed and the themes integrated and revised through mutual discussion by the entire research group. At that point the findings were structured according to Bronfenbrenner’s (1986) theory and in a final step the report was prepared. See Figure 1 for the stages of the coding process.

### Trustworthiness

We engaged in a number of procedures to ensure the trustworthiness of the findings (Krefting, 1991). The interview transcripts and manuscript were reviewed by an outside auditor, who agreed that the findings were faithfully represented in the manuscript. A participant in the research study also reviewed the manuscript, and replied, “I read it and found it excellent. Really good work. I believe that it addresses the responses of the focus group.”

The members of the research team, all women professionals, reflected on their own positions with respect to the research process and topic. The first author, a European-American woman originally from the United States, has lived in Central America for over 20 years. She is deeply involved in documenting the experiences of women around the world and has reflected that her lens privileges the strengths of women, rather than their lack of agency. The second author is Honduran with more than 15 years of experience in the management and planning of social, recreational, and educational activities for training young people from diverse cultural backgrounds. She has worked in psychosocial intervention on issues of education, employability, youth, gender, and social vulnerability in international cooperation organizations. The third author is Honduran, passionate about education, mother of two children who, through her own experience, has witnessed the challenges that women must overcome to grow in the professional world and is a faithful witness of how education changes lives. The fourth author is Honduran, an educator for more than 25 years at different educational levels; “that experience has allowed me to know that if women are given participation and empowerment, it affects the quality of their lives and their families.” The fifth author is a Bolivian who has lived in Honduras for the last 22 years. She has raised a family in that country, has two children and dreams of making Honduras a country with many opportunities for women and their families. She has worked for many years on projects to improve the food and nutritional security of children in the Central American region. The sixth author, a Honduran with more than 15 years of experience as a Human Resources professional, wrote, “I believe that all of us have the opportunity to generate positive changes in our environment. I firmly believe in the role of women making organizations warmer and more harmonious places.”

## Results

According to the Bronfenbrenner ecological model, the chronogram is an essential system to track the relation between person and environment over time. Because the participants constructed coherent narratives of the past, present, and future, significant statements and coding are presented separately for each of three time periods: before attending the university, while attending the university, and predicted/desired futures.

### Prior to Enrolling at the University

**FIGURE 2:**
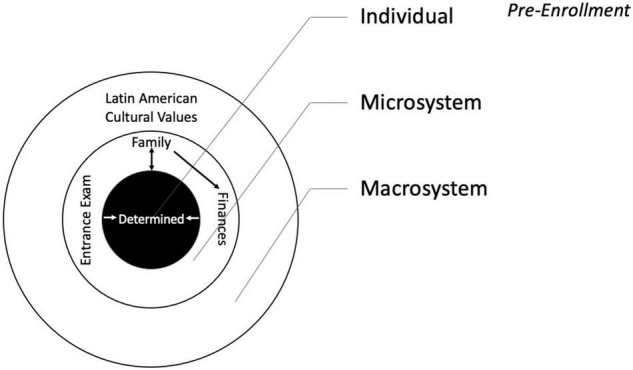
Depicts the ecological systems of the student prior to her studies at the university.

Even before enrolling at the university, participants expressed determination. Parents were most often supportive of their daughters’ plans. ‘‘Jessica^1^ “, a third-year student, explained, “my mother has always helped me and when I told her I wanted to come here, there was no hesitation, but on the contrary she encouraged me more.” Sometimes extended families made sacrifices to pay the student’s tuition. Victoria, a fourth-year student had the support of not only her parents, but also grandparents, aunts, and uncles, who even sold property to fund her education. Some messages from family members referred to greater difficulties for women in agriculture than for their male counterparts. Soraya, a second-year student said, “most people told me that [agriculture] was men’s work” and Amatista said, “my parents told me… that one would have to work and it would be more difficult for a woman.” Juliana, a third-year student said, “neither my mother nor my father helped me, they didn’t want me to be so far from home…. I had to find transportation to get here myself, but I said, ‘I’m going’ and I bought what I needed.” Thus, the primary microsystem of students before entering the university was their families, who provided both support and cautions about potential difficulties. Other challenges prior to attendance including passing the entrance exam and acquiring the finances necessary for tuition. Figure 2 presents a graphic of the ecological systems of the student pre-enrollment.

### The University Experience

#### The Woman in Agriculture

**FIGURE 3:**
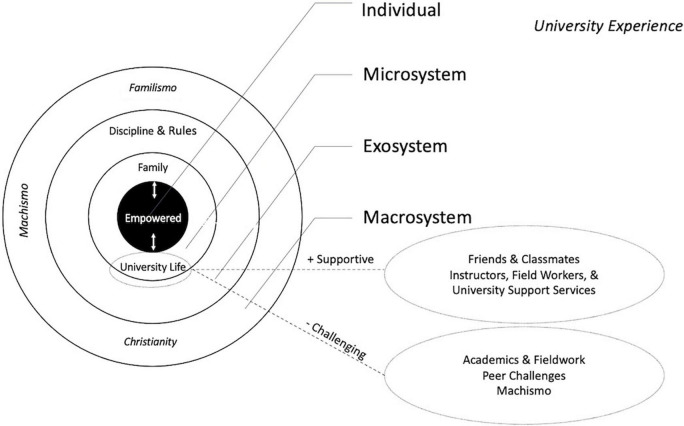
Depicts the ecological systems of the student during her studies at the university.

Figure 3 depicts the ecological systems of the student during her studies at the university. At the center of Bronfenbrenner’s schema (Bronfenbrenner, 1977) is the individual person. Participants agreed that the personal qualities necessary for success were to be focused, prepared, respectful, persistent, proud to be a woman, positive, and able to confront intrapersonal challenges, including self-doubt and uncertainty. A sense of self-efficacy was central. A second-year student, Amatista, said, “[We have to] take off that blindfold that there are things that women can’t do,” and “if I persist, I can achieve what I want.” A third-year student, Gloria, said, “Here, with dedication, one can always move forward,” and “if you set your mind to it, you can always achieve it.” Juana of the fourth year said, “Everything is a matter of organization, and if I want to I can,” and Tutis, also in the fourth year, said, “There is nothing that, with effort, cannot be done; there are no stupid human beings, only undisciplined ones.” Many described an empowerment process, including increased discipline, and self-efficacy from the first to fourth year of study. Ana, a fourth-year student, said, “Before, I was very insecure, I can’t do it, not this, always I was like that, but even in fieldwork, I learned to know my capabilities, to exceed my expectations.” Sometimes lapses in self-confidence were met with determination or by support from friends. Julieta, a third year student, said, “I was saying [to myself] ‘yes, I can’, this has been a challenge for me, telling myself every day, ‘I can.”’ Jenny had support from her woman friend, “you can, you can get through this, you can.”

Noteworthy was the personal growth mentioned by many participants. Amatista said this most succinctly, “I think that one grows as a person during these four years.” Soraya replied, “I grew as a person…..[I became] more responsible, and accustomed to making decisions for myself without depending on others. For me this was a great leap forward.” Victoria said, “being here at the university makes you mature,” Both Rubi and Tutis expressed what has been referred to as a growth mindset (Dweck, 2008), “I always try to be a better person….not only as a student, but in my relations with others, and in that way I think I can achieve my dreams,” and “there is nothing that one cannot achieve with effort.”

#### University Life

University life was the richest and most salient of the components of students’ lives, and comprised six themes, (1) university structure and discipline, (2) academic challenges, (3) institutional support from fieldworkers, instructors, and university counseling center, (4) peer support from classmates and friends, (5) peer challenges in the social environment (e.g., gaining social acceptance), and (6) machismo.

The strict rules and discipline imposed on students’ lives was seen as both a valuable tool for growth, and as an irritating constraint on autonomy. Stephany from the first year said, “I think I have progressed because one learns to organize one’s time. With 10 min free time one does a mountain of things. And the discipline as well, one knows what time they have to be there and at [this university] one has to arrive 5 min early.” Jeysul of the third year said, “I have learned a mountain…. discipline, that I can’t just make excuses.” “In my case I had to self-discipline better,” said Gloria. Silvana said, “studying and discipline were always a bit difficult for me.” Amatista described how her sleeping and studying routines were altered by the university rules, “I used to go to sleep at 8 in the evening and wake up at midnight to study until 4 am, and then sleep another hour and wake up again; this was my study habit and here you have to go to sleep at 11:30.” Soraya gave an example of the limits placed on laboratory use, “We want to experiment more and have the space and create our future,” but it was against the rules that dictated specific hours for laboratory work. Or phrased differently by Aysha, “I think, yes, we can make our own decisions, but based in the regulations.”

Academic classes and practica (fieldwork) were sometimes a challenge, but students reported receiving help from multiple people, including instructors, other students, and fieldworkers. Rubi expressed help she received from a number of sources, “Always instructors tried to take me aside to ask if there was something I didn’t understand in class, if I had questions to take advantage of [their help] and also my classmates also help me and they ask me if I know if we have homework, and if they can help me with homework or study habits in order to understand and succeed.” Jenny described her response to difficult classes, “you have in your mind that this class is difficult [one that can lead to you failing out], and I’m going to fail it, but no, no class is that difficult, that we are all capable of learning this material.” Skarleth pointed out that if you are too embarrassed to ask the instructor something the fieldworkers answer questions, “the truth is that they are very kind and they say, ‘come’ and they begin to explain, and because they are in the fields, they know a great deal, and they explain it.” Here [at the university] they give you all the resources you need to succeed….for example, they give you the time, the hours to study, tutorials…. I think that here they give you the confidence to come and be able to achieve all your goals,” said Jenny.

Faculty, staff, the counseling center, and a religious ministry provided emotional support as well as academic support to students. Jenny underwent a personal crisis while a student at the university. “People from the religious ministry, the counseling center, many times, no matter the hour, when I was crying and feeling bad, they came and opened up, we would talk, and they would try to see what I could do to feel better.” Jessica said, “I can speak to this because [two women from the counseling center] have been there in my moments of crisis; last year in a difficult moment without the help of those two, I would not have been able to control it; the help has been enormous.”

Peer support from classmates, roommates, and friends was vital to students’ social and emotional well-being. Soraya claimed that her roommate was a pillar of support, “because it is with her that I share experiences and… if one feels bad in the night or begins to think about things, because one is always going to miss the family or something bad will happen that is very difficult, well, she is always there to listen. In praising help from instructors, Aysha pointed out that, “A while ago, I had a family problem and other than my roommate and three classmates that I consider my best friends [they also supported me]. So, for Aysha the primary sources of support were her roommate and friends. Ana, in her 4th year at the university, received support from a [male] friend, “well, I wasn’t friends with him in the first or second years, but when we were in the third year of studies, he saw I was having difficulties with some things, and he was very available and attentive and he has helped me a lot.” A fourth year student underscored the importance of friendship, “My best friends during these four years I suppose are [always] going to be my best friends and they are people in who I confide my experience and I don’t hesitate to ask them something I don’t know and I am not afraid to be myself.”

The women sometimes faced challenges in the social environment. “[I had challenges] in social relations, relating to people, because at the beginning I didn’t know anyone, and then I came to know many people,” said Ana, a first-year student. Cultural differences were sometimes problematic, “how to live in a different country, different from your country. Even though we are all Latinas, we have differences. This is something shocking, both the food and the culture,” said Sandy. “I had to get to know new people, other cultures,” said Juana, a fourth-year student. Soyaya, in her second year, said, “[When I arrived] I was not very focused on land or agriculture, and they were making fun of me, and one of the seniors came over and said, “You can do it; if I can, why not you?” In her advice to incoming students, Jessy admonished, “Sometimes you will be bullied, and things like that, but be strong and persist.”

Machismo on the part of classmates was a common experience at the university. Tutis expressed this strikingly, “Ugly as it sounds [new women students] are entering the lions’ den; they are the fresh meat.” She continued, “[My advice is] don’t let them sweettalk you, because all lions want to take advantage of you.” A milder form of machismo was reported by the first- year student, Gaby, “the boys from the other years, they view us like objects, they don’t see us as people, with feelings.” Machismo was most prevalent during fieldwork that often required physical strength. According to Aquira her male classmates would complain that she was small and thin. Skarleth pointed out that men paired with women in work groups complained, “[women] don’t work, arrive late and [men] do the work.” Sometimes tasks were assigned according to gender, “they divide us by work, girls in light work and boys in more arduous work.” On the other hand, women were sometimes preferred as co-workers for academic tasks, “I think that in the academic domain, in class, [men] give us more responsibility, because they prefer working with women…. Because they are a little more distracted let’s say, and it’s more difficult for them,” said Elizabeth. The women reported determination in confronting machismo, “We are more determined when we set a goal, it is women who give advice here to men on how to behave, how to study. Although we are a minority I think we are better,” said Sarai, a first-year student. And in response to men who said, “You women are not good for anything, not for falling in love or taking to bed,” Jeysul replied, “I came to study, not to fall in love or to go to bed with anyone.” Soraya said, “Even though we are not men, we have the same strength as them, everything depends on our willpower and the effort we make.”

#### Family Relations

Although all students resided at the university, their families continued to play a prominent role in their lives. Families provided emotional and financial support, but also were a source of stress and responsibility. Victoria expressed the centrality of family, often called *familismo*, in Latin American culture, “I believe that the fundamental pillar in my life is my family.” In response to a question about who has helped them achieve their goals, the most prevalent answers were parents, family, and siblings. Jeysul a third-year student said, “Every time I feel like I can’t do it…… I call my mother…. It’s like ‘mamá, I feel like I can’t, I feel tired,’ and she is like, ‘lie down and sleep…. drink some sugar water;’ for me my mother is that help, she has always been that support for me.” Many students reported missing their families while attending the university. According to Soraya, “you are always going to miss your family.” “It is difficult because you don’t have your brother, even to fight with or to bother you, but you have to overcome that.” Advice from family members often took the form of proverbs: “The immensity of sacrifice matches the immensity of success,” was the pithy saying in Skarleth’s family. Being apart from one’s family could also be stressful when the family experienced problems, For Ana, in her fourth year of university, the biggest challenge was having an aunt who was sick with terminal cancer, and she feared the worst with every phone call. Tutis, also in her fourth year of studies, said, “you are part of the family problems, but at the same time not [present], so all those things, those conflicts, the news, those impulses where you can’t do anything because you’re so far away…. I’m the oldest daughter so I get four versions of the problems at home – my mother, my father, and my siblings.”

#### Faith and Religion

Although religion was not a prominent theme, it was mentioned as a support system in each focus group. Sarai used the common phrase “primero Dios [God willing] I will graduate,” as an expression of hope. As reported above, Jenny received support from the church ministry. Gloria, a third-year student noted that going to church helped her deal with stress. And Soraya, a second-year student was grateful to God for the opportunity to study, “[it] was an opportunity that life has given us, that God has given us.”

### Aspirations for the Future

**FIGURE 4:**
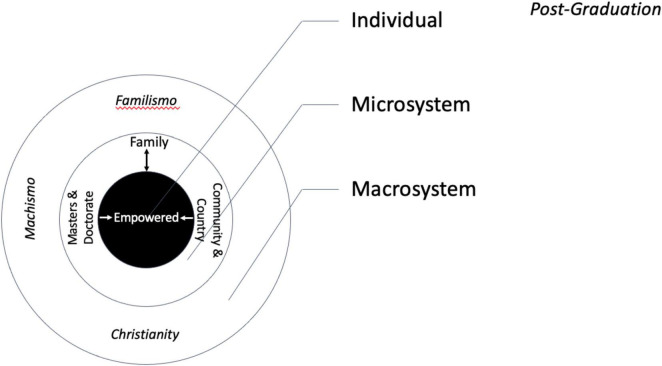
Graphically depicts the anticipated systems of the student post-graduation.

A sense of empowerment was highlighted in the students’ future goals and aspirations. Figure 4 graphically depicts the anticipated systems of the student post-graduation. The widely-shared immediate goal was to graduate from the university. Julieta, a third-year student said, “right now my goal is to graduate.” Beyond graduation, two objectives were prevalent: to acquire further education, including a masters and doctorate, and to use the skills acquired to help others. Victoria was one of several respondents who planned to earn masters and doctoral degrees. She was inspired by fieldwork because, “it helped me to understand the focus on research, I loved it and want to continue with it.” A desire to help others in one’s country of origin was prominent. For Rubi, a first-year student, her main target was the street children of her country, “In my country there is a problem with children who live on the street because they don’t have parents; these children have to seek food alone; [this university] has given me [the means] to do something for these children and to help them.” Jessica also focused on the children of her country, “My idea is that when I return to [my country] to motivate young people to get better grades.” Jessy (second-year student) said, ‘my country is essentially agrarian; I want to return to my country to help people.” Elizabeth explained that it was not just theory that students learned at the university, but, “we can solve the problems of our countries, we can help agriculture in our countries to become more sustainable, and we can feed the world because that is what they are teaching us. Juana said, “My country has the potential, in which one can work to help people with scarce resources, giving them the knowledge one has obtained here.” Like many students, Julieta was focused on her family, “I want to help my family, my mother, to see the new horizons that I have.” For Tutis, expanding her father’s business was important, “It had never been my intention to work for a big company, I prefer to start from the bottom-up…. [as the oldest child] I feel a great responsibility I want to bring animal husbandry to my father’s business.” However, even personal goals sometimes merged with community well-being. According to Stephany, “I want to establish my own business, generating employment in the community.”

## Discussion

Empowerment has been defined as, “the ability to exercise choice” (Kabeer, 1999, p. 436). Women studying at the agricultural university asserted their empowerment, not only in the title quote, “there is nothing I cannot achieve,” but also in similar statements, such as, “we can, there is no limit.” Their advice for incoming students was, “they also have the capacity, like us, to come here and confront the daily challenges and forge ahead.” According to Kabeer (2005), there are three critical components to empowerment: Resources, agency, and achievement. The valuable education women are receiving is a critical resource that will serve them in the future. Their sense of agency is highlighted in their repeated references to self-efficacy, and the participants, having demonstrated academic achievement through success at the university, aspire to greater achievements in the future. Thus, the process of empowerment was evidenced by students’ gains in all three components of the concept.

Although the data for this study were collected at a single point in time, students’ reflections on their previous and anticipated experiences allow a glimpse at the process of change, labeled as the chronogram by Bronfenbrenner (1977). Before arriving at the university their personal qualities of determination and perseverance were already present. Some women reported taking the entrance exam more than once and making intense efforts to attain scholarships and the necessary funding. Their major microsystem was the immediate family who provided support, but sometimes cautioned that being a woman studying agriculture would be arduous.

At the university, the personal quality of persistence despite obstacles was prominent. A major theme was the expression of self-efficacy, confidence in one’s capability to carry out actions and achieve goals. Although self-efficacy, per se, was not specifically encompassed in the original Women’s Empowerment in Agriculture Index (WEAI), it was incorporated as a part of agency in a revised version of the WEAI (Martinez et al., 2021, and can be seen as central to women’s empowerment at the university. Self-efficacy theory, proposed by Bandura (1977) posits that self-efficacy is promoted by mastery experiences, vicarious experiences, supportive others in the environment, and specific internal states. Mastery experiences were prevalent among the women students; for example, Gloria said, “and last trimester I was able to do it better.” Although vicarious experiences were not frequent, several third-year students reported looking to graduates of the university for advice and support. Having social support from others at the university, including classmates, instructors, fieldworkers and counselors was a major theme that emerged from the analysis. Jessica reported helping her classmates by urging them to be calm (and confident). Soraya pointed out that the female students “have serenity when it comes to doing the homework.” In sum, it seems that the conditions that promote self-efficacy were present in the environment.

The salient microsystems during the university experience included the social environment of peers, classmates, and friends, the classroom experiences, fieldwork, university services such as the counseling center, and continued relations with families. Among the themes that were identified in those microsystems were challenges with respect to academics and fieldwork, peer challenges as such gaining social acceptance and dealing with cultural differences, and machismo. Support was provided by the institution from fieldworkers, instructors, and university counseling center and also from peers, especially classmates and friends. Determination, self-efficacy, will-power, and persistence were strategies that helped them cope with the challenges, as well as the utilization of helpful others in their social networks. Machismo, in particular, was addressed by maintaining a positive attitude, recognizing women’s strengths, and gaining the respect and affection of their male classmates through their own competence and interpersonal skills.

The strict regulations and discipline dictated by the university might be considered an exosystem in Bronfenbrenner’s terms (Bronfenbrenner, 1977), given that students did not participate in their formulation. Those stringent regulations impacted classwork, fieldwork, and interpersonal relationships. Students saw both advantages and disadvantages to the rules. Although the rigid rules helped them to set priorities and manage their time, they constrained, to some extent, students’ autonomy in pursuing their own academic (and other) goals.

Widely-shared cultural values are considered by Bronfenbrenner (1977) as part of the macrosystem. For the students in our study, the Latin American cultural features of *familismo* (familism), machismo, and religious faith were evident and as pointed out by Vélez-Agosto et al. (2017) instantiated in their everyday experiences.

*Familismo* or familism is a central feature of Latin American culture, defined as, “[placing] the family ahead of individual interests and development. It includes many responsibilities and obligations to immediate family members and other kin” (Ingoldsby, 1991, p. 57). The three dimensions of familism — family as referents, family as support systems, and familial obligations (Sabogal et al., 1987) — were highlighted by the participants. Among many others who used the family as a referent, Jessica said, “I am the first in my family to have an international scholarship.” Extensive familial support, the second characteristic, was not only emotional, but also tangible, as illustrated in the response of Victoria’s extended family to her tuition needs. They rallied to provide the funds, even selling property to raise money. The responsibility that the women felt toward their families of origin was unmistakable. Tutis was distraught when she could not address the problems of her family, given her physical distance from them. In her statement that, “I’m the oldest daughter so I get four versions of the problems at home,” she not only referred to her position in the family but her obligations to them. Familism, especially family obligations, may be more salient in the lives of women than in their male classmates (Campos et al., 2014).

Women’s roles in Latin America are also shaped by the concept of marianismo, the view that the ideal woman should emulate the Virgin Mary (Castillo et al., 2010). A central feature of marianismo is that women are expected to be the pillar of the family and to assume the primary responsibility for family well-being. Although familism is generally considered a protective factor for Latinos and Latin Americans (Corona et al., 2017) and some forms of marianismo may be empowering (Maegli et al., 2022), responsibilities to the family may weigh more heavily on women students, resulting in more complex and mixed consequences for their well-being and academic achievement (Vega, 2014; Zhu et al., 2021).

While machismo, an exaggerated, hypersexual, and aggressive masculinity that is based in men’s power over women and often involves subordination of women, is recognized as a core part of Latin American culture (Toro-Alfonso, 2009), it may be more glaring and weighty in the agricultural educational environment. During fieldwork students are called to carry 100 pound sacks, to wield machetes, and to manage large farm animals. Tutis admired a female classmate, who although tiny, could carry as much as her male classmates. She concluded that, “And I think that [this university] should focus on the idea that we can all do it, that we [women] have the ability.” Agricultural studies, because of the physical work involved, may pose additional challenges compared to other fields of study, such as engineering, in which women face being a minority.

Although widespread, machismo is not uniform nor universal among all communities or individual men in Latin America (Arciniega et al., 2008; Sara-Lafosse, 2014; Gibbons and Luna, 2015). In our study, Gaby emphasized individual differences among her male classmates, “it depends on the personality of the [men] whether they help you or not.” Jenny noted their assistance, “the support of my [male] classmates takes place not only in the educational realm and learning by doing, but also in supporting [my] emotional well-being.” Cinthia said, “Well, they help us a lot in fieldwork when they see, for example, that we can’t do something …. Or they think it is very dangerous for us to do it. They are considerate at times.” And Soraya noted that her male classmates could be protective, “When we leave the university on the weekend, and we are with our male classmates, the men are the ones who take care of us, ‘she is my colleague, my classmate,’ and if something happens they defend us, so one feels safe with them.” These two examples illustrate another side to machismo, labeled “caballerismo’ by Arciniega et al. (2008). The benevolent dimension of machismo dictates that men should be chivalrous; they should protect and defend women and their families (Arciniega et al., 2008). Overall, however, there were many everyday examples of machistic behaviors, and the second-year student Elizabeth commented, “I believe that we still live in a machistic society, that puts aside women and believes that because of strength or other abilities that we don’t have, that we are less, and I believe that this has to change.”

Christianity is the dominant religion among Latin Americans. In 2020, over 80% of Latin Americans claimed a Christian faith (Statistica, 2020). Moreover, the majority of people in most Latin American countries say that religion is very important in their lives (Pew Research Center, 2014). Although religion and faith were present in the group discussions of students, they did not assume a primary role. In fact, most of the codes for religion came from short phrases that allude to God (e.g., *Gracias a Dios, Si Dios quiere, Primero Dios*), and are colloquialisms, rather than indicators of religiosity. For the university students, attending church was an extracurricular activity and served as a support group. These findings might reflect the current decline in religiosity, especially among middle class youth, in Latin America (Rodríguez Cuadros, 2018). Nonetheless, it is critical to mention that for two students, Gloria and Victoria, faith in God was a major support. Gloria said, “it helped me a great deal to go to church….it is a moment for the person and God.” Victoria said, “you feel as if God is always with you and that he is going to influence the decisions you make.” Even though religion *per se* may not have been a dominant force in students’ lives, some of the basic tenets of Christianity seemed to permeate their thinking. Many anticipated a life of service to others and described gender roles derived from Christianity.

There were dynamic interactions among the ecological systems of the women students. Their roommates, classmates, friends, and instructors encouraged them in both academics and fieldwork. Instructors and fieldworkers sometimes served as proxies for parents and older siblings, dispensing support and advice. Support from others was seen as essential to women’s achievement and thriving at the university. Ana in her first year said, “I think that here in this environment I have learned that we need other people, that we need one another; on my part my classmates have helped me, have helped me a great deal, teachers also, the counseling center of the university.” Reliance on others to thrive may be a universal need, but may also be exaggerated by cultural collectivism in Latin America (Hofstede, 2001; Krys et al., 2022; Salvador et al., 2022).

Everyday experiences of differential gender roles, reciprocity, and close familial relationships reflected cultural values of Latin America. The contrast between fieldwork, where men often disparaged women’s work and the classroom, where they preferred working with women and often assigned them leadership roles may relate to the familism and gender role ideologies prevalent in Latin America. The outside fieldwork may be analogous to the public sphere where men often dominate and can assert their power, whereas the classroom may resemble the private sphere, in which Latin American women have exercised relative power (Stevens, 1973; Smyth, 2008; Maier, 2010). The marianismo ideology positions women as the pillar of the family and women’s leadership in the academic work groups may stem from that underlying belief system.

With respect to their futures, the female agricultural students saw their horizons expanding. They anticipated further education, establishing their own businesses, as well as contributing to their families, communities, and countries. Some wished to travel abroad and explore other nations and continents. The women’s desire to make a contribution, both to their families and their countries may stem, in part, from the familism and marianismo values, both of which emphasize service to others.

The students in our study are well-positioned and empowered for their futures. As accomplished graduates, they will be able to serve as role models for other women working in agriculture (Stern et al., 2016). As role models they may encourage more women to engage in agricultural work, moving Latin America beyond the current 20% of female agricultural workers (FAO, 2011). Because of the students’ extensive fieldwork, they have practical as well as theoretical knowledge to share. Moreover, they are practiced at addressing machismo, a skill that will undoubtedly be useful in their futures. Those skills and activities position women with an agricultural education to contribute to the United Nations SDGs, not only with respect to gender equality (SDG5), but also to the elimination of poverty (SDG1), zero hunger (SDG2) and good health and well-being (SDG3).

## Limitations and Future Directions

This study documented the experiences of a group of women students attending an agricultural university, but it cannot be generalized to other situations or conditions. And despite attention to the trustworthiness of the findings and the employment of reflexivity, member checking and auditing, the investigators were prepared to notice the growth and transformation of students, rather than their mistakes and missteps. The researchers’ shared commitment to women’s empowerment may have inadvertently influenced the focus group discussions, as well as the coding and interpretation of the findings. Although a sense of empowerment was clearly expressed in the focus group discussions, the specific personal and environmental factors fostering empowerment are unknown. According to the participants, the keys to success included physical and emotional effort, maintaining a positive attitude, not trusting too readily, relying on friends and family, recognizing that this is not a “feminine” life, and keeping a focus on the future. More research is needed to further document the preconditions for empowerment and to explore the women’s future challenges and successes as professional agriculturists in Latin American cultures. In addition, to fully understand gender interactions and processes at the university, the views of men and persons who are non-binary with respect to gender should also be queried about their gender attitudes and the factors that promote equity across all gender categories.

The findings have implications for university policies and initiatives. The recruitment of women students could be prioritized to achieve gender parity in enrollment. Moreover, women students relied heavily on social support from many others – including male and female classmates, faculty, and fieldworkers – to achieve empowerment. Therefore, initiatives to maintain or increase sources of support are essential. Workshops or programs on gender equity might be initiated, perhaps aimed primarily at men (Acheson and Kelly, 2021). Universities might celebrate women’s accomplishments, for example, on International Women’s Day, through their newsletters and announcements. A code of conduct could be established, expanded, or promoted to prohibit machistic or sexist behavior.

In sum, a number of ways have been proposed to empower women in agriculture. Among those are initiating agricultural development projects (Johnson et al., 2018), establishing women’s agricultural cooperatives, (Pastran, 2017), integrating gender issues into agricultural research, (Meinzen-Dick et al., 2010), and providing extension courses aimed at women participants (Diaz and Najjar, 2019). However, it is clear that higher education in agricultural and rural development can provide a transformational experience to women, empowering them to feed the world.

## Data Availability Statement

The datasets presented in this article are not readily available because the transcripts of the focus groups contain potentially identifiable data. Requests to access the datasets should be directed to JG, judith.gibbons@slu.edu.

## Ethics Statement

The studies involving human participants were reviewed and approved by Human Subjects Research Board, Hope College. The participants provided their written informed consent to participate in this study.

## Author Contributions

JG designed the research study and wrote the manuscript, participated in the construction of the interview questions, the coding of the blinded transcripts, and the data analysis. ZE-F, AM-A, GM-F, IV-M, and AA-S participated in the construction of the interview questions, the conducting of the focus groups, the coding of the blinded transcripts, the data analysis, and reviewed the manuscript for accuracy and completeness. ZE-F led the local team with respect to the research conduct and coding. AM-A served as the liaison with the university administration. All authors contributed to the article and approved the submitted version.

## Conflict of Interest

The authors declare that the research was conducted in the absence of any commercial or financial relationships that could be construed as a potential conflict of interest.

## Publisher’s Note

All claims expressed in this article are solely those of the authors and do not necessarily represent those of their affiliated organizations, or those of the publisher, the editors and the reviewers. Any product that may be evaluated in this article, or claim that may be made by its manufacturer, is not guaranteed or endorsed by the publisher.

## Footnotes

1
*All names are pseudonyms chosen by the participants.*
